# Suspected fenugreek (*Trigonella foenum-graecum* L.) toxicosis in a herd of Saskatchewan beef cattle

**DOI:** 10.1177/10406387241307974

**Published:** 2025-01-09

**Authors:** Vanessa E. Cowan, Roman V. Koziy, Laurie Zemlak, John R. Campbell

**Affiliations:** Departments of Veterinary Biomedical Sciences, Western College of Veterinary Medicine, Saskatoon, Saskatchewan, Canada; Prairie Diagnostic Services, Saskatoon, Saskatchewan, Canada; Peak Veterinary Health, Moose Jaw, Saskatchewan, Canada; Large Animal Clinical Sciences, Western College of Veterinary Medicine, Saskatoon, Saskatchewan, Canada

**Keywords:** cattle, fenugreek, fenugreek poisoning, neurotoxicity, peripheral neuropathy, plant poisoning

## Abstract

An apparent outbreak of fenugreek forage toxicosis occurred in a beef cattle herd near Moose Jaw, Saskatchewan in February–May 2022. The herd had consumed fenugreek hay from late fall to early winter. Clinical signs included various degrees of weakness, ataxia, knuckling, walking on hocks, and recumbency. All adult cattle in the herd eventually died or were euthanized. Feed analysis did not reveal nutritional deficiencies or mycotoxin contamination. Liver mineral and vitamin status of affected animals did not indicate any consistent abnormality. The last live cow in the herd was presented to a veterinary teaching hospital for evaluation and subsequent postmortem examination. Major postmortem findings included emaciation, and sciatic nerve and spinal cord axonal degeneration. Histologic examination of the sciatic nerve showed Wallerian-like axonal degeneration, increased Schwann cell nuclei, and endoneurial fibrosis. Histologic examination of the spinal cord showed infrequent myelin sheath dilation and digestion chambers within white matter. These results are consistent with other reports of natural and experimental outbreaks of fenugreek poisoning in livestock. To our knowledge, fenugreek toxicosis has not been reported previously in Canada. We conclude that caution should be taken when feeding fenugreek hay to cattle.

Fenugreek (*Trigonella foenum-graecum* L.) is an annual legume forage that was introduced to Canada in the 1990s.^1^ It has been proposed as an alternative forage for livestock in the semiarid Western Canadian prairies^8^ and as a rotational break crop.^13^ In addition to its low water requirements, fenugreek is rich in nutrients and bioactive compounds.^2,5,20^ Purported benefits of feeding fenugreek to cattle are improved protein digestibility and reduced greenhouse gas emissions.^20^ Fenugreek is also used as a bloat-free forage in cattle due to the presence of condensed tannins.^20^ In lactating dairy cows, fenugreek has been shown to improve milk characteristics.^15^ However, a different study showed that fenugreek inclusion as haylage had lower feeding value due to lower dry matter intake and lower milk yield.^6^ In beef heifers, fenugreek extract with organic trace minerals improved average daily gain and efficiency with lower cost per kilogram of gain.^9^ However, a study of fenugreek extract in beef bulls found no effect on the starter through finisher phases.^19^ Additionally, beef steers fed barley-supplemented fenugreek silage had similar dry matter intake, average daily gain, and feed efficiency compared to barley-supplemented alfalfa silage.^11^ None of these studies reported abnormal effects in cattle. In Saskatchewan, fenugreek is an uncommon crop; in 2016, 1,137 ha (2,809 ac) of fenugreek were grown in Saskatchewan.^16^

In contrast to the numerous apparent agricultural and livestock performance benefits of fenugreek, there is a small body of evidence describing peripheral neuropathy of ruminants grazing fenugreek forage. A 2023 report from Spain described proprioceptive deficits and hindlimb weakness in 100 of 400 cattle following 1 mo of consumption of fenugreek hay^12^; 60% of affected animals died or were euthanized. Histologically, peripheral nerves from affected animals had Wallerian degeneration and vacuolation of myelin sheaths. This syndrome was replicated experimentally in sheep, but not goats. “Fenugreek staggers” is described as a locomotor syndrome of grazing ewes in Australia.^7^ Additionally, the earliest reports of fenugreek toxicosis in livestock come from the mid-20th century and describe locomotor and myodegeneration in cattle and sheep.^3,4^ In all published reports, a toxic principle has not been identified. We retrieved no cases of fenugreek poisoning in livestock in Canada in a search of Google, PubMed, CAB Direct, and Scopus, using search terms “fenugreek poisoning livestock Canada”, suggesting that this condition has not yet been reported in Canada.

An outbreak of peripheral neuropathy occurred in a beef cattle herd in southern Saskatchewan beginning in late February 2022, which was assumed to be caused by the consumption of fenugreek hay. The outbreak took place in a small herd of commercial beef cattle (2 bulls, 36 cows and bred heifers). The cattle were fed monensin-medicated commercial pellets and free-choice fenugreek hay, buckwheat hay, and straw beginning in November 2021 and throughout the winter feeding period. Because the fenugreek hay was offered free choice, an estimate of intake could not be made. Cows and bred heifers received 1.4 kg (3 lb) of pellets/head/d in the late fall, 2.8 kg (6 lb)/head/d in the winter, and 3.6 kg (8 lb)/head/d in very cold weather. The bulls received 2.8–3.6 kg (6–8 lb)/head/d in the later fall and winter, and 5.4 kg (12 lb)/head/d at the time of the outbreak. The cattle had reportedly been in good body condition prior to the outbreak.

The first apparent case was in an older farm bull that was stiff and reluctant to move on 2022 Feb 22 (day 0). Within days, despite removal of the suspect pellets, the bull progressed to severe lameness and weakness, which included dropped hocks (Fig. 1). The herd veterinarian suspected gastrocnemius rupture and recommended euthanasia (day 6). No postmortem examination was performed on the bull, as it was assumed that this was the only animal affected, and the assumed problem was trauma related.

**Figures 1–4.**
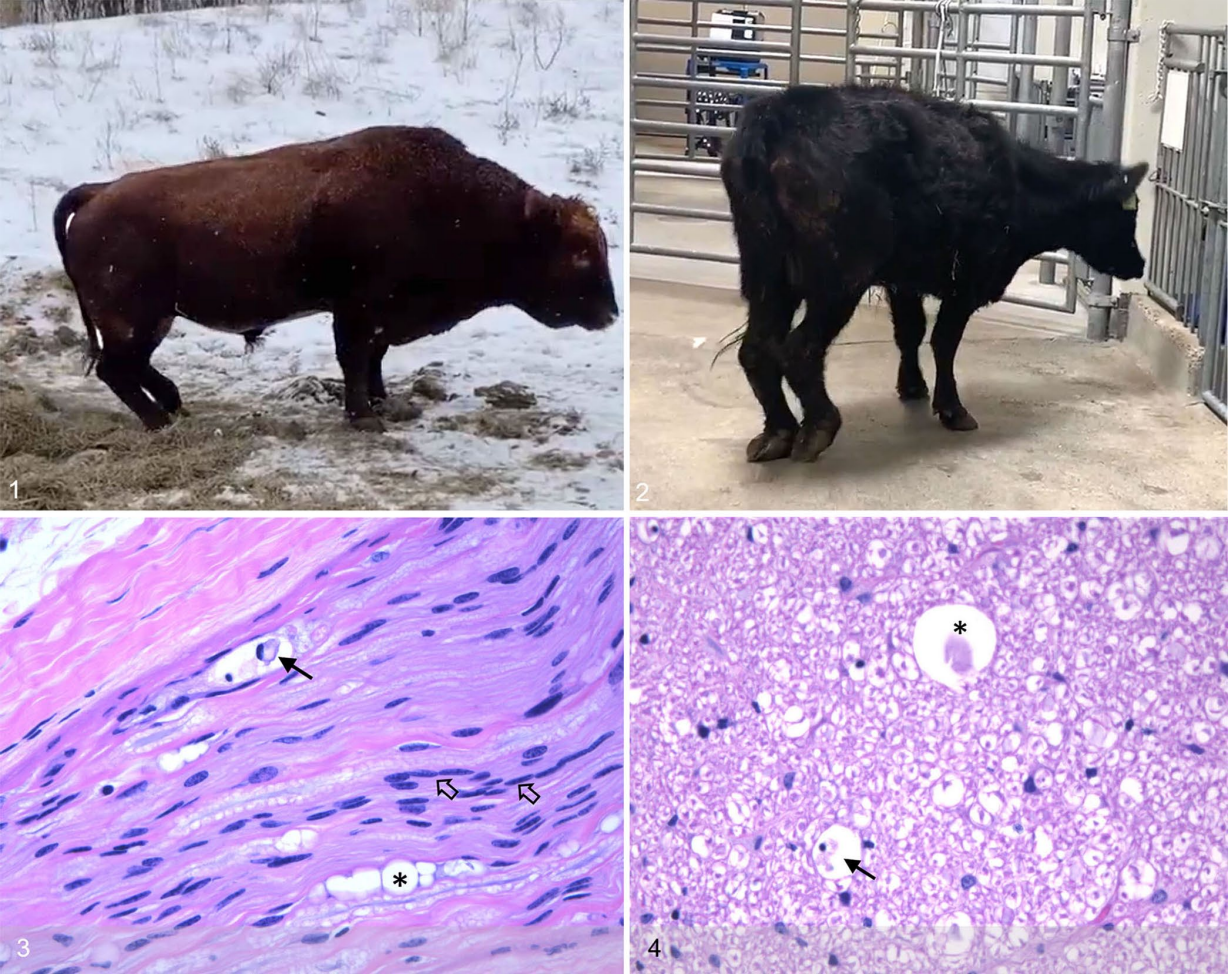
Outbreak of peripheral neuropathy in cattle following months of consuming fenugreek (*Trigonella foenum-graecum* L.) hay on a commercial beef farm in southern Saskatchewan. **Figure 1.** First reported animal (a bull) affected on-farm in February 2022. **Figure 2.** The last live animal (cow 5) from the herd was presented to the Western College of Veterinary Medicine Large Animal Clinic in May 2022 for assessment. Note knuckling over in both hindlimbs. **Figure 3.** Sciatic nerve from cow 5. Wallerian degeneration characterized by dilation and vacuolation of myelin sheaths (*) and presence of digestion chambers with macrophages (black arrow). Bands of Schwann cells (Büngner bands) are indicative of regeneration attempts (open arrows). H&E. **Figure 4.** Spinal cord from cow 5. Wallerian-like degeneration with dilation and vacuolation of myelin sheaths containing swollen axons or spheroids (*) and digestion chambers with macrophages (arrow). H&E.

By day 11, the herd veterinarian estimated that one-third of the herd was affected with a similar presentation to the bull. Clinical signs included weakness, knuckling on fetlocks, wide-based stances, and recumbency. It was noted that, initially, the hindlimbs were more severely affected than the forelimbs. The cattle also were reported to have an increased respiratory effort. A field postmortem examination was performed on an affected cow (cow 1) by the herd veterinarian. No gross abnormalities were identified. The gastrocnemius muscles were intact. The cow was ~8-mo pregnant with good body condition and fat stores. Liver from cow 1 was submitted to a veterinary diagnostic laboratory (Prairie Diagnostic Services [PDS]; Saskatoon, Saskatchewan, Canada) for trace mineral and vitamin E evaluation (Suppl. Table 1) to test for nutritional deficiencies, specifically nutritional myopathies (i.e., vitamin E and/or selenium deficiency). Trace minerals were quantified in liver by inductively coupled plasma–mass spectrometry. Vitamin E was quantified in liver by high-performance liquid chromatography (HPLC).

Throughout March and April, samples from various affected cattle were sent to PDS for clinical pathology, serum chemistry, trace mineral and heavy metal status, and vitamin status (Suppl. Tables 1, 2). Autopsies were not performed on all cattle. Sera from heifers 1 and 2 and cow 1 were submitted to PDS for CBC analysis (Advia 2120i hematology system; Siemens) and chemistry panel analysis (cobas c 311 analyzer; Roche) to evaluate underlying infectious or inflammatory processes. There were no apparent patterns in the few abnormalities observed in serum chemistry. Trace minerals were quantified in serum for heifers 1 and 3 and cow 2, and quantified in liver for heifers 1 and 3 and cows 1, 3, and 4. Trace mineral concentrations were within RIs according to published reference values,^14^ except copper deficiency in heifer 1 (i.e., serum Cu <0.5 ppm; RI: 0.6–1.50 ppm). However, the liver copper concentration of heifer 1 was in the high-normal range.^14^ The neurotoxic metals (Pb, As, Th, Cd) were present in below-toxic concentrations. Vitamin concentrations were interpreted according to published reference values.^14^ Vitamin E was quantified in liver for cow 1 and heifer 1—both results were considered high-normal. Vitamin D was within normal limits for the 3 animals tested (heifers 1 and 2 and cow 3). There was no common trend in trace mineral or vitamin status in the cattle tested; therefore, the results of this testing ruled out nutritional deficiencies or metal toxicities as a cause of disease in the herd.

In addition to sampling animal tissues, feed samples were collected and sent for analysis. The suspect commercial pellets and new pellets were sent to PDS in mid-March for mycotoxin analysis (by liquid-chromatography mass spectrometry; Suppl. Table 3) due to concerns for potential mycotoxicosis. The concentration of mycotoxins in both types of pellets was considered background contamination. Due to the initial suspicion that the cattle were experiencing ionophore toxicosis, a sample of the commercial pellets were sent to the Animal Health Laboratory at the University of Guelph (Guelph, Ontario, Canada) to determine ionophore concentrations by HPLC. The concentration of monensin, salinomycin, and narasin were 93 µg/g, <1.0 µg/g, and <1.0 µg/g of pellets, respectively. The reported limit of quantitation for these analytes is 1 µg/g. The monensin concentration was higher than the label value of 66 µg/g; however, this was not considered to be high enough to cause toxicosis (calculation in Suppl. Text). Additionally, there were no cardiac lesions consistent with monensin toxicosis. Overall, the results of the feed testing ruled out mycotoxicosis or ionophore toxicosis as the cause of the outbreak.

The herd continued to deteriorate throughout the rest of March and April. By day 30, there were 4 cows dead, 5 down, and 15 with similar clinical signs. Calving occurred during this time, and the calves were unaffected (Suppl. Video). Heifer 3 died while she was being moved to the sick pen. The field veterinarian performed a postmortem examination and reported some heart dilation and petechiation of the heart, but no visible endocarditis. The only other abnormalities noted were hepatic scarring (suspected to be chronic) and adhesions on the diaphragm. Multiple cows that could not rise were euthanized due to poor prognosis. Liver, heart, tongue, and lung were collected from multiple cattle (heifer 3, cows 3 and 4) during on-farm autopsy and subsequently submitted for histopathology. In general, histologic evaluation of the tissues did not reveal any significant morphologic changes. Myonecrosis was observed in the tongue of cow 4, but it was unclear whether this finding was related to the animal’s clinical signs. Mild nonsuppurative hepatitis was observed in heifer 3; however, this was attributed to a resolved bacterial infection. There were no significant findings in any tissues from cow 3, or in other tissues from cow 4 and heifer 3.

By day 40, the entire herd was affected. The Western College of Veterinary Medicine (WCVM) Disease Investigation Unit was recruited at this time. Samples of the commercial pellets, hay, and fenugreek hay were sent to Cumberland Valley Analytical Services (Waynesboro, PA, USA) for basic nutritional analysis (Suppl. Table 4). The nutritional content of each feed was considered adequate for beef cattle. Given the results of the feed analysis and trace mineral and vitamin status, the overall plane of nutrition for the herd appeared adequate.

On day 67, the producer brought the last live cow in his herd, cow 5, to the WCVM Veterinary Medical Center Large Animal Hospital (Saskatoon, SK, Canada) for evaluation (Fig. 2). The owner reported that she had been in good condition before neurologic signs began. On presentation, this cow had a body condition score (BCS) of 1, was markedly ataxic and could only walk a short distance. The cow had a base-wide stance, her hocks would drop low to the ground while attempting to walk, she would also frequently knuckle over on her hind legs. Her vision appeared to be normal as she moved into a pen; however, her instability precluded detailed physical examination. The serum chemistry panel had evidence of hepatocellular injury and cholestasis and slight hyperglobulinemia (Suppl. Tables 2, 5). The minimal mature neutrophilia suggested mild inflammation, and thrombocytosis was considered to be reactive due to inflammation and/or catecholamine release. The cow was euthanized due to poor prognosis and submitted for postmortem examination.

Gross examination of cow 5 revealed emaciation (BCS 1 of 5) and mild-to-moderate pulmonary edema. The bone marrow fat percentage was 14.4%, indicative of depletion of fat reserves and emaciation (RI: 70–90%).^10^ Interpretation of the clinical and autopsy findings indicated that the poor condition of cow 5 was a result of the neuropathy, rather than another condition.

Sections of sciatic nerve and spinal cord from cow 5 were collected and processed routinely for histopathology. Microscopic evaluation of the sciatic nerve revealed multifocal moderate Wallerian degeneration of axons with eosinophilic debris and rare degenerate macrophages (Fig. 3). Additionally, there were increased Schwann cell nuclei (bands of Büngner) with mild-to-moderate fibrosis in the endoneurium, indicating a chronic process. Microscopic evaluation of the spinal cord revealed rare dilation of myelin sheaths within the white matter with occasional swollen axons (Fig. 4). Degenerate cells with hyperchromatic nuclei and eosinophilic flocculant debris were consistent with digestion chambers. The subarachnoid space had locally extensive hemorrhage. An incidental finding in the cerebrum was a single protozoal cyst with no associated tissue damage or inflammation, consistent with *Sarcocystis* spp. Other regions of the brain examined (cerebral cortex, basal nuclei, thalamus, hippocampus, midbrain, medulla, and cerebellum) had no significant histologic changes.

Other tissues of cow 5 assessed histologically included the lung, spleen, a regional lymph node, heart, kidney, liver, adrenal gland, ileum, colon, tongue, and urinary bladder. The lung contained an abscess that was considered to be an incidental finding. Similarly, protozoal cysts within myocardiocytes with mild myocarditis was considered to be consistent with *Sarcocystis* spp. and were considered incidental findings. The kidney, liver, adrenal gland, ileum, colon, tongue, and urinary bladder had no significant histologic lesions identified.

Extensive diagnostic testing ruled out underlying infectious disease, trace mineral and vitamin deficiencies, ionophore toxicosis, mycotoxicosis, or metal toxicosis. The mixed breed and age of the cattle also ruled out genetic abnormalities. Further, the calves born during the outbreak were unaffected. Nitropropionic acid from *Astragalus* spp. plants has been associated with Wallerian degeneration of the sciatic nerve in cattle^18^; however, the cattle in our case were not grazing rangelands and did not have access to *Astragalus* spp. plants. In regions with significant *Astragalus* spp. populations, nitropropionic acid poisoning should be considered in outbreaks of peripheral neuropathy of grazing livestock.

A presumptive diagnosis of fenugreek toxicosis was made after comparing the histologic findings and clinical signs in this case with the few reports available and through testing that ruled out other possible causes.^7,12^ Specifically, the herd-level presentation of progressive hindlimb ataxia and axonal Wallerian degeneration of the sciatic nerve supported this diagnosis. This presentation was similar to both a natural outbreak of fenugreek toxicosis in cattle and an experimental feeding trial in sheep in Spain,^12^ which described proprioceptive deficits, hindlimb weakness, and recumbency. That report examined the peripheral nerves of several affected animals and documented the most severe lesions in the sciatic nerve. Additionally, pregnant cattle affected in the natural outbreak did not experience reproductive problems, which is consistent with our case. The producer in our case did not report any increase in fetal loss, dystocia, or neonatal calf disease. The calves born to affected cattle did not develop neurologic disease within 4 mo of observation of the herd.

In contrast to the report from Spain, there were a few differences in presentation. The duration of ingestion before the onset of disease was substantially shorter in those animals compared to our case (3–4 wk in cattle and 3 mo in sheep in Spain, versus several months in our case). The difference in the exposure-to-onset interval may be related to the amount of fenugreek fed, local differences in climate and fenugreek growth, and possible differences in the amount of the unknown toxin. The estimated total intake in the affected cattle in Spain was 100 kg fenugreek straw per animal.^12^ Additionally, only 25% of the cattle herd in the Spanish outbreak was affected as opposed to the entire herd in our report. This observation may be related to the longer duration of fenugreek consumption in the outbreak that we have described here. Due to the limited reports of fenugreek toxicosis in livestock, the pathogenesis of toxicosis, including any dose-response relationship, is unknown. The producer in our case had fed fenugreek hay to his cattle for many years without obvious clinical problems occurring until 2022. We speculate that the drought experienced by the region in the summer of 2021^17^ may have resulted in increased amounts of the unknown toxin within the plant. It is also possible that the producer was feeding a higher percentage of fenugreek hay due to the unavailability of other forages.

If the use of fenugreek is limited as a forage for cattle, fenugreek toxicosis is a rare syndrome in livestock. However, based on our findings and in the 2023 report from Spain,^12^ the morbidity and mortality rate of the outbreaks can be high. Therefore, caution should be taken when feeding fenugreek hay to cattle. More research is required to characterize the toxicity of fenugreek before it can safely be fed to cattle as an alternative forage. Our report highlights the importance of histologic examination of the nervous system in cattle with hindlimb ataxia, as Wallerian degeneration of axons supported the diagnosis in our case.

## Supplemental Material

sj-pdf-1-vdi-10.1177_10406387241307974 – Supplemental material for Suspected fenugreek (Trigonella foenum-graecum L.) toxicosis in a herd of Saskatchewan beef cattle Supplemental material, sj-pdf-1-vdi-10.1177_10406387241307974 for Suspected fenugreek (Trigonella foenum-graecum L.) toxicosis in a herd of Saskatchewan beef cattle by Vanessa E. Cowan, Roman V. Koziy, Laurie Zemlak and John R. Campbell in Journal of Veterinary Diagnostic Investigation
