# Supplementary material for: Suspected fenugreek (Trigonella foenum-graecum L.) toxicosis in a herd of Saskatchewan beef cattle
Source: J Vet Diagn Invest. 2025 Jan 9;37(2):329–33. doi: 10.1177/10406387241307974 (PMC11719421; doi:10.1177/10406387241307974)
Supplement: sj-pdf-1-vdi-10.1177_10406387241307974 – Supplemental material for Suspected fenugreek (Trigonella foenum-graecum L.) toxicosis in a herd of Saskatchewan beef cattle [file sj-pdf-1-vdi-10.1177_10406387241307974.pdf]

JVDI: Supplemental material

Cowan VE, et al. Suspected fenugreek (*Trigonella foenum-graecum* L.) toxicosis in a herd of Saskatchewan beef cattle

Supplemental Text. Calculation for monensin exposure.

LD_{50} of monensin in cattle: 22 mg/kg[1]

Adult cow:

Assumed body weight: 600 kg

Maximum pellet consumption: 3.6 kg (8 lb)

Monensin concentration: 93 mg/kg

93 mg/kg × 3.6 kg pellets = 335 mg monensin

335 mg ÷ 600 kg body weight = 0.558 mg/kg body weight dose received

~39× lower than LD_{50}

Adult bull:

Assumed body weight: 1,000 kg

Maximum pellet consumption: 5.5 kg (12 lb)

Monensin concentration: 93 mg/kg

93 mg/kg × 5.5 kg pellets = 512 mg monensin

512 mg ÷ 1,000 kg body weight = 0.51 mg/kg body weight dose received

44× lower than LD_{50}

Supplemental Table 1. Liver trace mineral and vitamin E concentrations (wet weight basis) of clinically affected cattle, by sample and sample month (2022).

	Heifer 1		Cow 1	Cow 2	Heifer 2	Cow 3	Heifer 3	Cow 4	Cow 5
	Serum	Liver	Liver	Serum	Serum	Liver	Liver	Liver	Liver
	March	March	March	March	March	April	April	April	May
Arsenic, ppb	0.912	6.50	6.18	0.972	1.46	15	9.72	13.4	12.8
Antimony, ppb	0.172	0.493	0.531	0.13	0.166	1.62	0.552	1.28	1.18
Barium, ppb	130	31.4	36.3	129	168	321	66.7	219	42.9
Beryllium, ppb	0.411	<0.004§	1.43	0.771	0.581	1.82	0.723	1.65	0.246
Bismuth, ppb	0.101	<0.001§	<0.001§	0.040	0.373	<0.001§	<0.001§	<0.001§	0.051
Cadmium, ppb	<0.006§	109	77.5	<0.006§	<0.006§	84.6	37.1	140	68.2
Chromium, ppb	7.62	<0.01§	132	6.52	5.74	39.5	21.5	32.5	70.7
Cobalt, ppb	1.19	98.3*	81	1.44	1.70	118*	89.1*	83.6	137*
Copper, ppm	0.383‡	139*	68.8	0.649	*0.536*	132*	163*	183*	174*
Iron, ppm	3.03[H]	138	145	3.76[H]	3.41[H]	112	85.6	80.8	81.2
Lead, ppm	NS	0.003	0.009	NS	NS	0.026	0.011	0.058	0.013
Magnesium, ppm	25.2	228	127	23.0	24.6	244	153	197	169
Manganese, ppm	0.006	1.92†	2.05	0.006	0.01	5.61	2.26	3.82	1.80†
Molybdenum, ppm	0.018	0.458	0.963	0.014	0.021	0.807	0.562	0.393	1.09
Nickel, ppb	8.47	<0.017§	49.6	9.02	16.0	40.9	8.88	21.9	24.6
Selenium, ppm	0.185	1.34*	0.780*	0.220	0.201	0.993*	0.781*	1.06*	1.11*
Strontium, ppb	91.3	18.8	60.4	54.3	130	217	53.9	128	65.0
Thallium, ppb	0.362	0.502	0.538	0.11	0.42	2.72	0.991	6.68	0.484
Tin, ppb	2.76	<0.007§	5.15	2.45	0.154	14.2	0.512	2.47	0.662
Vanadium, ppb	1.17	2.6	3.12	1.02	2.35	58.5	6.06	41.6	8.96
Vitamin E, ppm	NA	92.9*	54.3*	NA	NA	NA	NA	NA	NA
Zinc, ppm	1.35	72.8	34.1	1.2	1.88*	122	82.4	42.3	57.5

NA = not analyzed; NS = not suitable. Superscript H indicates hemolysis.

* High-normal (considered over-supplementation but within the range of normal).

† Marginal (considered suboptimal nutrition but within the range of normal).

‡ Deficient.

§ Below the limit of detection.

Supplemental Table 2. Blood chemistry panel and vitamin D results from clinically affected beef cattle, by sample and sample month (2022).

	Heifer 1	Cow 2[H]	Heifer 2[H]	Cow 3	Cow 5	
	Serum	Serum	Serum	Serum	On cells	
	March	March	March	April	May	RI
A:G ratio	1.17	1.03	1.14	0.950	0.640	0.550–1.19
Albumin, g/L	41.0*	38.0	40.0*	38.0	34.0	32.0–38.0
Anion gap, mmol/L	26.0	24.0	26.0	26.0	22.0	17.0–29.0
AST, U/L	118	96.0	108	129	236*	42.0–131
Bicarbonate, mmol/L	22.0	23.0	24.0	25.0	23.0	17.0–33.0
Direct bilirubin, µmol/L	0.70	0.40	0.800	0.60	0.80	0.0–3.0
Indirect bilirubin, µmol/L	0.70	1.60	2.50*	1.00	1.20	0.0–1.5
Total bilirubin, µmol/L	1.40	2.00	3.30	1.60	2.00	1.0–5.0
Calcium, mmol/L	2.66*	2.53	2.59	2.64*	2.48	2.21–2.61
Chloride, mmol/L	96.0	97.0	95.0	100	98.0	91.0–104

CK, U/L	683*	198	381*	244	170	64.0–344
Creatinine, µmol/L	87.0	101*	109*	106*	72.0	49.0–95.0
GGT, U/L	13.0	9.00†	11.0†	19.0	47.0*	12.0–39.0
GLDH, U/L	27.0	8.00	28.0	7.0	249*	7.00–36.0
Globulin, g/L	35.0	37.0	35.0	40.0	53.0*	32.0–52.0
Glucose, mmol/L	4.10	3.40	3.40	3.20	4.20	1.60–4.40
Magnesium, mmol/L	1.08	1.02	0.94	1.00	0.86	0.81–1.13
Phosphorus, mmol/L	1.79	1.43†	2.07	1.75	2.30	1.45–2.59
Potassium, mmol/L	5.40*	6.50*	5.50*	6.50*	4.80	3.70–5.30
Sodium, mmol/L	139	137†	139	144	138	138–148
Total protein, g/L	76.0	75.0	75.0	78.0	87.0	68.0–87.0
Urea, mmol/L	4.90	5.20	5.70	3.70	4.10	3.50–10.3
Vitamin D, ppm‡	0.033	0.035	NA	0.039	NA	0.02–0.06

A:G = albumin:globulin ratio; AST = aspartate aminotransferase; CK = creatine kinase; GGT = gamma-glutamyl transferase; GLDH = glutamate dehydrogenase; NA = not available. Superscript H indicates slight hemolysis noted for cow 2 and heifer 2.

* Higher than RI.

† Lower than RI.

‡ Separate analysis (HPLC).

Supplemental Table 3. Mycotoxin concentration (mg/kg) of the pellets present when the outbreak started and the reformulated pellets.

Mycotoxin	Old pellets	New pellets
3+15-acetyl-DON	<16.0*	<16.0*
Aflatoxin B1	<0.400*	<0.400*
DON	74.5	72.3
DAS	<16.0*	<16.0*
Fumonisin B1	18.4	20.8
Fumonisin B2	6.27	6.00
HT-2 toxin	9.51	9.70
Nivalenol	<16.0*	<16.0*
Ochratoxin A	9.83	5.09
T-2 toxin	7.51	6.37
Zearalenone	7.66	<4.00*
Ergocornine + ergocorninine	3.40	<2.00*
Ergocristine + ergocristinine	13.0	27.2
Ergocryptine + ergocryptinine	5.39	4.96
Ergometrine + ergometrinine	<2.00*	<2.00*
Ergosine + ergosinine	5.95	19.7
Ergotamine + ergotaminine	2.36	2.19
Total ergot alkaloids	30.1	54.1

DAS = diacetoxyscirpenol; DON = deoxynivalenol.

* Below the limit of detection.

Supplemental Table 4. Feed analysis results of pellets, hay, and fenugreek hay. Feed analysis was conducted by Cumberland Valley Analytical Services (Waynesboro, PA, USA).

	Pellets	Hay	Fenugreek hay
Dry matter	89.2	83.7	88.5
Moisture	10.8	16.3	11.5
Carbohydrates			
Crude Fat, %DM	3.85	1.92	1.19
Ethanol soluble carbohydrates, %DM	NA	1.8	NA
Starch, %DM	NA	2.2	1.2
Water soluble carbohydrates, %DM	NA	2.2	5.0
Energy & index calculations			
Total digestible nutrients, %DM	63.7	50.2	47.3
Net energy lactation, Mcal/lb	0.65	0.50	0.47
Net energy maintenance, Mcal/lb	0.68	0.43	0.37
Net energy gain, Mcal/lb	0.42	0.18	0.13
Metabolizable energy, Mcal/lb	1.08	0.81	0.75
Neutral detergent fiber digestion rate, Kd	NA	3.1	4.0
Relative feed value	NA	76	NA
Non-fiber carbohydrates, %DM	32.1	17.7	22.5
Ethanol soluble carbohydrates, %DM	NA	4.0	NA
Water soluble carbohydrates, %DM	NA	4.4	6.2
Fiber			
Acid detergent fiber, %DM	21.5	45.1	53.5
Neutral detergent fiber, %DM	39.9	66.2	66.0
Lignin, %DM	5.36	9.02	10.3
Minerals			
Ash, %DM	9.61	7.25	6.96
Calcium, %DM	1.29	0.83	1.17
Copper, ppm	56	NA	NA
Iron, ppm	692	NA	NA
Magnesium, %DM	0.30	0.29	0.25
Manganese, ppm	175	NA	NA
Phosphorus, %DM	0.48	0.20	0.04
Potassium, %DM	0.84	1.99	1.19
Sodium, %DM	0.08	NA	NA
Zinc, ppm	226	NA	NA
Protein			
Ammonia, CPE	NA	1.26	NA
Crude protein, %DM	15.8	11.0	3.8
Soluble protein, %DM	6.0	3.6	1.9
ADF protein, %DM	1.05	1.91	1.34
NDF protein, %DM	1.32	3.97	1.53
Rumen degradable protein, %DM	NA	7.3	2.8

%DM = percentage of feed on a dry matter basis; Kd = rate of digestion; Mcal/lb = megacalories per pound; NA = not available.

Supplemental Table 5. CBC results from a clinically affected 2-y-old Aberdeen Angus × heifer (cow 5) hospitalized in the Veterinary Medical Center Large Animal Clinic.

	Result
Erythrogram	
RBCs, $\times 10^{12}$/L	6.84
Hemoglobin, g/L	112
Hematocrit, L/L	0.302
MCV, fL	44.1
MCH, pg	16.4
MCHC, g/L	373
RDW, %	15.9
Morphology	Anisocytosis (1+) echinocytes 1 (2+)
Leukogram	
Eosinophils, $\times 10^9$/L	0.24
Lymphocytes, $\times 10^9$/L	3.12
Monocytes, $\times 10^9$/L	0.56
Neutrophils†, $\times 10^9$/L	4.08
WBCs, $\times 10^9$/L	8.00*
Thrombogram	
Platelets, $\times 10^9$/L	772*
Clumped	Yes
Plasma proteins	
Fibrinogen, g/L	6.00
Total protein, g/L	86.0
TP:Fb	14:1

MCH = mean corpuscular hemoglobin; MCHC = mean corpuscular hemoglobin concentration; MCV = mean corpuscular volume; RDW = width; TP:Fb = total protein to fibrinogen ratio

* Higher than RI.

† Segmented neutrophils.

Supplemental Video. Multiple affected cattle with normal calves.

References

1. Van Vleet JF, et al. Clinical, clinicopathologic, and pathologic alterations in acute monensin toxicosis in cattle. Am J Vet Res 1983;44:2133–2144.
